# Odontogenic cystic lesion segmentation on cone-beam CT using an auto-adapting multi-scaled UNet

**DOI:** 10.3389/fonc.2024.1379624

**Published:** 2024-06-12

**Authors:** Zimo Huang, Bo Li, Yong Cheng, Jinman Kim

**Affiliations:** ^1^ School of Computer Science, The University of Sydney, Sydney, NSW, Australia; ^2^ State Key Laboratory of Oral & Maxillofacial Reconstruction and Regeneration, Key Laboratory of Oral Biomedicine Ministry of Education, Hubei Key Laboratory of Stomatology, School & Hospital of Stomatology, Wuhan University, Wuhan, China

**Keywords:** bioengineering, cone-beam 3D CT, convolutional neural network, deep learning, informatics

## Abstract

**Objectives:**

Precise segmentation of Odontogenic Cystic Lesions (OCLs) from dental Cone-Beam Computed Tomography (CBCT) is critical for effective dental diagnosis. Although supervised learning methods have shown practical diagnostic results in segmenting various diseases, their ability to segment OCLs covering different sub-class varieties has not been extensively investigated.

**Methods:**

In this study, we propose a new supervised learning method termed OCL-Net that combines a Multi-Scaled U-Net model, along with an Auto-Adapting mechanism trained with a combined supervised loss. Anonymous CBCT images were collected retrospectively from one hospital. To assess the ability of our model to improve the diagnostic efficiency of maxillofacial surgeons, we conducted a diagnostic assessment where 7 clinicians were included to perform the diagnostic process with and without the assistance of auto-segmentation masks.

**Results:**

We collected 300 anonymous CBCT images which were manually annotated for segmentation masks. Extensive experiments demonstrate the effectiveness of our OCL-Net for CBCT OCLs segmentation, achieving an overall Dice score of 88.84%, an IoU score of 81.23%, and an AUC score of 92.37%. Through our diagnostic assessment, we found that when clinicians were assisted with segmentation labels from OCL-Net, their average diagnostic accuracy increased from 53.21% to 55.71%, while the average time spent significantly decreased from 101s to 47s (P<0.05).

**Conclusion:**

The findings demonstrate the potential of our approach as a robust auto-segmentation system on OCLs in CBCT images, while the segmented masks can be used to further improve OCLs dental diagnostic efficiency.

## Introduction

Odontogenic Cystic Lesions (OCLs) are one of the most common pathologic entities in the jaw region with a high incidence rate. OCLs can be divided discretely into cysts and tumors. According to Johnson et al. (1), the most common types of OCLs are the radicular cyst (RC), dentigerous cyst (DC), and odontogenic keratocyst (OKC), respectively. Among these three cysts, the RC and DC are benign and non-invasive, whereas OKC is highly likely to reoccur and exhibit locally aggressive behavior and malignant transformation (2). Among the OCL tumors, ameloblastoma (AM) is the most frequent one, which is a benign lesion with a slow growth rate, and it can invade local tissues such as the mandible and maxilla (3). Treatment of AMs generally requires radical resection, with a longer follow-up period compared to other tumors (4). On the other hand, cysts like OKCs and DCs are often treated with curettage and enucleation, and sometimes DCs can be treated by marsupialization to allow the tooth to be maintained (5). However, on CBCT images, tumors like AMs and cysts like OKCs or DCs exhibit similar characteristics, making it difficult to distinguish between them. Therefore, differentiating between tumors and cysts, as well as identifying specific subtypes is crucial because different lesions require distinct treatment plans. Accurate segmentation of OCLs ensures that appropriate areas have been highlighted which can help with differentiating lesions on images.

**Figure 1:**
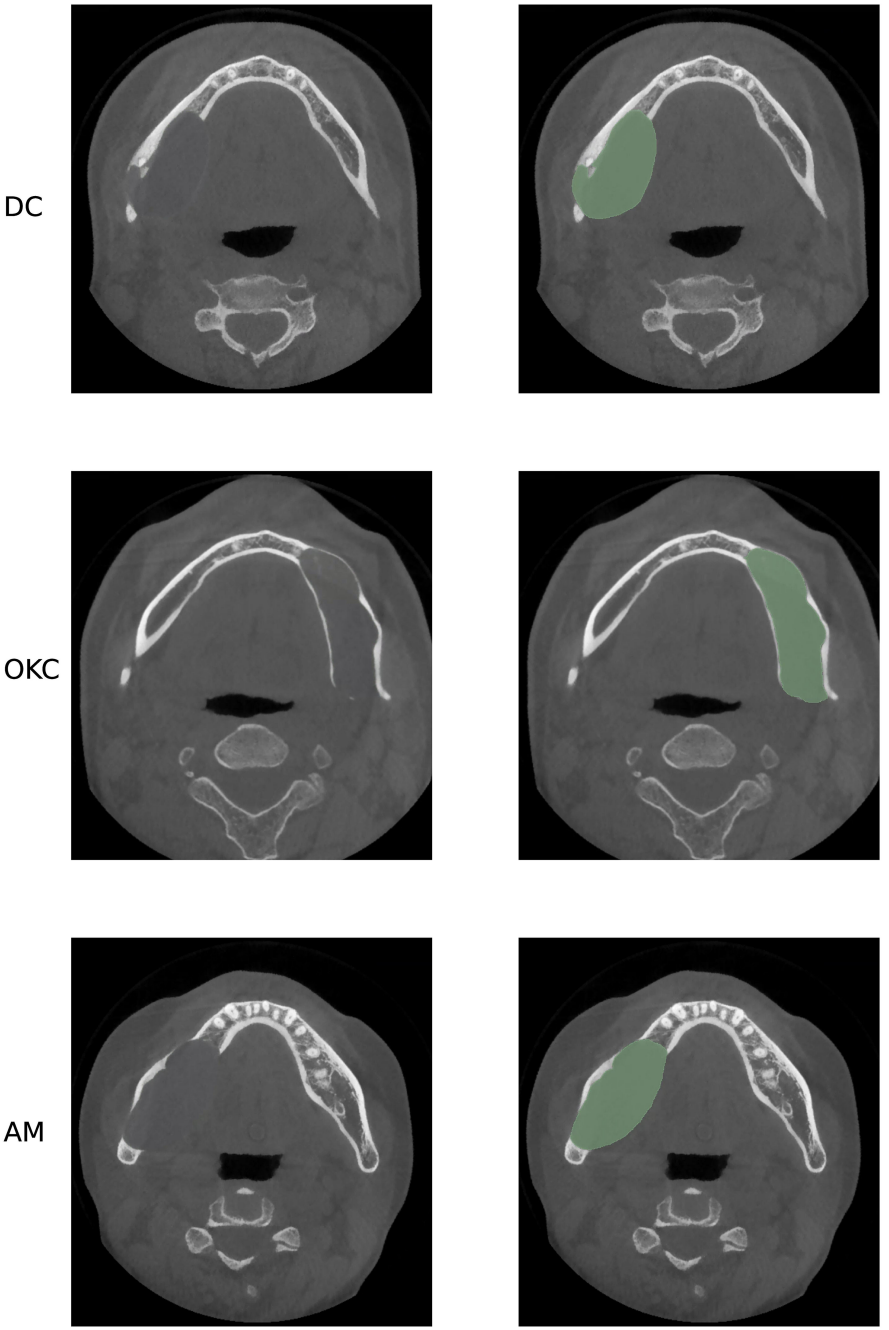
Axial CBCT presents three different lesions, all in the mandibular area, from top to bottom: DC, OKC and AM. As presented, these three lesions have similar size, location, and shape, which may cause misdiagnosis in real practice.

Radiographic imaging examinations are vital for patients with odontogenic cystic lesions, notwithstanding that histopathological findings are the gold-standard diagnostic criteria. However, histology examination needs to be performed after the surgery with the entire specimen excised whereas radiographs are non-invasive and can be obtained beforehand (6–8). Cone-beam computed tomography (CBCT) is an innovative imaging technique that employs divergent X-rays that form a cone shape. The adoption of CBCT has enhanced the accuracy of diagnosing dental diseases, especially OCLs that are commonly found inside the jawbones and are challenging to diagnose without imaging techniques that can demonstrate the 3-dimensional information compared to normal X-rays or Panoramic Radiographs (9, 10). However, accurate diagnosis of the lesion subtypes is often difficult as different odontogenic cystic lesions may share similar features on CT imaging. For example, as presented in **Figure 1**, AMs can be confused with large OKCs and the unicystic variant of AMs often associated with the crown may mimic DCs (11). Given the similarities between the various subtypes of OCLs, radiologists must examine several imaging features, such as the presence of the lesion, its size, location, and internal structures as whether the lesion includes any crowns or roots, to make an accurate diagnosis, treatment response monitoring and planning therapeutic interventions. For these features, accurate segmentation of cysts and tumors is an essential step as the segmentation output can be used to derive imaging features necessary for subtype diagnosis. However, segmentation is a complex task that is usually done manually, which is tedious, error-prone, and time-consuming (12). Therefore, an automated segmentation mask generated by a computer-aided system can be an effective tool to extract imaging features related to subtype diagnosis, thereby improving diagnostic accuracy, and reducing time consumption.

Over the past few years, a great interest has been focused on deep learning-based methods, which have been extensively used for solving complex problems in medical radiology (13–15). Deep convolutional neural networks (CNNs) have become increasingly utilized in medical image segmentation (16–21) with the introduction of UNet and its variants (22–25), great improvement has been made in segmentation tasks. In recent years, there has been an increase in the use of deep learning methods for various dental imaging problems. For instance, in 2016, researchers used UNet for dental anatomical segmentation on bitewing image modality (26). Similarly, for more complex tasks such as tooth segmentation, the use of FCN-based U-Net was proposed for the dental panoramic radiograph segmentation task (27). Additionally, researchers applied the same U-Net structure to segment panoramic radiographs and achieved successful results with 88% Dice in segmenting apical lesions, which is a sub-disease within OCLs (28). Several well-developed CNN architectures, such as FCN and UNet (29, 30), have been applied in dental image segmentation tasks resulting in state-of-the-art performances (31). For imaging analysis tasks developed for OCLs, researchers mainly focused on developing reliable methods to diagnose these cysts and tumors (32–35). However, segmenting multiple types of cyst-like lesions within CBCT images remains a challenging task since there is no public dataset and the annotation process is hard to complete. Therefore, our research is the first to propose a deep learning method optimized for OCL segmentation on large curated and annotated CBCT data specifically focused on DC, RC, OKC and AM. Furthermore, we compared the diagnostic accuracy of the proposed model with that of senior and junior oral and maxillofacial surgeons.

In this paper, we proposed an automated segmentation of odontogenic cystic lesions on CBCT images. To tackle the issue of size variability among different subtypes, we incorporated a multi-scale module and an auto-adapting schema into the UNet architecture. Our primary contributions are summarized below:

1. We proposed a multi-scale module that operates at different image scales to guide the model to extract information under different scales and overcome the problem of subtype size variation by preserving all scales’ features regardless of their size.
2. We proposed an auto-adapting technique that uses training outcomes to re-adjust the size of input images, discarding unnecessary background information and retaining only the essential information for further training to help the model focus on the lesion.
3. Through extensive experiments on a curated CBCT dataset, we demonstrate the effectiveness of our proposed network for the supervised segmentation of lesions. We further demonstrate that our method can positively affect diagnostic assessments by junior and senior surgeons through a comparison experiment that asks them to diagnose the OCLs using CBCT images with and without the help of segmentation labels.

## Materials and methods

### Data preparation

CBCT images (in DICOM format) from a total of 300 patients (51 DCs, 53 RCs, 117 OKCs and 79 AMs), were included in this study from the Stomatological Hospital of Wuhan University, China. Full ethical approval was obtained. The patients were selected retrospectively based on specific inclusion criteria. The criteria required clear pathological diagnosis, complete image data, and exclusion of images with excessive artefact, poor image quality, or beyond the field of view. The images with multiple lesions and the coexistence of numerous pathological types were also excluded. All cases were confirmed by histopathology examinations after surgery, and personally identifiable information was removed. Three radiological experts manually annotated the pixel-wise label map of each image using 3D Slicer and stored it as a pair of MHD files for both the image and the label. During the model training process, 20% of the images were randomly selected to be the test set and the rest 80% were used for training.

### Model overview

**Figure 2:**
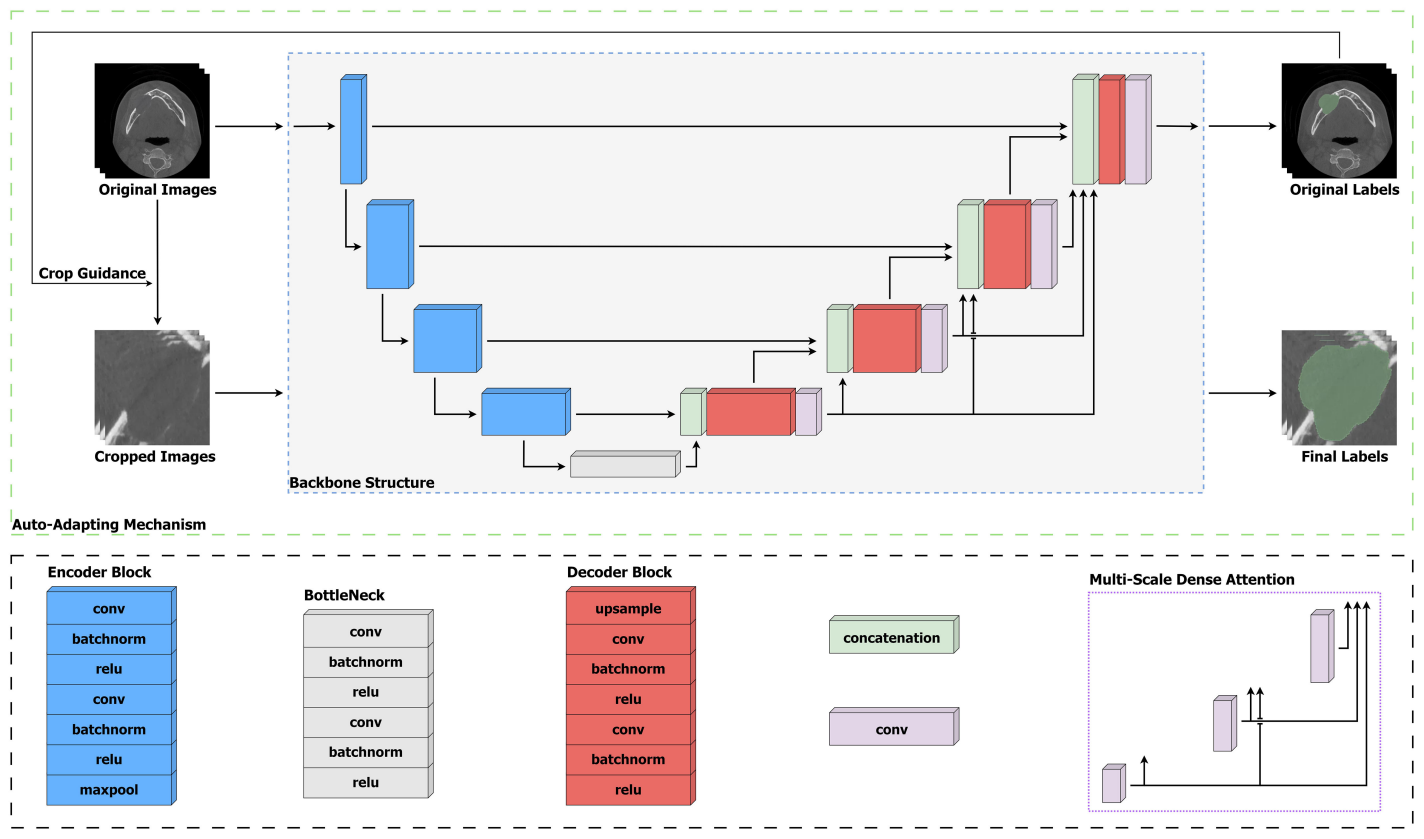
Structure of our proposed OCL-Net. To address the inter-class variation problem among different subtypes, the late four scales employ convolution layers to generate multi-scale feature maps to enforce the learning process. The gradually trained prediction mask is sent through the auto-adapting connection to be scaled up and help centralize the input images.

An overview of our proposed OCL Network (OCL-Net) is illustrated in **Figure 2**. Similar to its backbone U-Net, it employs an encoder-decoder structure which is commonly used in medical imaging segmentation. The encoder structure consists of five parts, with each part representing a different scale, and is implemented by a convolutional block together with a max-pooling layer for down-sampling. In each convolutional block, two convolutional layers are used, and each layer is followed by a batch normalization layer and a Rectified Linear Unit. The decoder followed a similar design structure with every max-pooling layer being switched to a transposed convolutional layer to up-sample the feature map back to the input size. To enhance the encoding and decoding process, skip connections are utilized between corresponding levels of the encoder and the decoder, allowing the network to retain crucial spatial information and gradient flow. These connections concatenate the feature maps from the encoder to those in the decoder, facilitating a more accurate reconstruction of the input data. Moreover, the integration of dropout layers after each max-pooling operation aids in mitigating overfitting, ensuring that the model generalizes well across different CBCT scans. In addition to the backbone structure, we have integrated two novel techniques, including a multi-scale dense attention module and an auto-adapting schema, into the network. The multi-scale dense attention module enhances spatial awareness by generating and integrating attention maps across different scales, allowing the model to focus on pertinent image regions progressively. This mimics the clinical process of interpreting CBCT scans from broad features to finer details. The auto-adapting schema dynamically adjusts the input images during training to eliminate redundant background information, centralizing the lesions for more effective learning. By cropping the images based on predicted labels, the model refines its focus, improving segmentation accuracy. The details of these two modules will be further explained in subsequent parts of this section.

### Multi-scale dense attention module

The four OCL subtypes have various positions and sizes, and to incorporate these different characterizations into our model, we integrate an adjusted multi-scale dense attention module to enhance the network’s spatial awareness. This follows the work of (36) who integrated multi-scale guided blocks to segment complex Pulmonary Fibrosis lesions, which have a set of similar imaging characteristics compared to OCLs. Specifically, we use a convolutional layer at the late four scales of the decoder to retrieve a spatial attention map which is used as a high-level attention signal to guide the learning of the following decoding process. The input of the decoder at each scale will be incorporated with all the attention maps generated in the previous scales. Therefore, the multi-scale module ensures that for every lower scale, the spatial information from all the previous higher scales will be preserved as an additional input.

In terms of architectural design, the multi-scale dense attention module employs a sequence of convolutional layers followed by normalization and activation functions to generate attention maps at each scale. Each attention map is produced by retrieving the output of a 1×1 convolution, enabling the model to emphasize or suppress features at different locations. The novelty lies in how these attention maps are recursively integrated: at each scale, the current attention map is concatenated with the feature map from the previous layers before being passed to the next convolutional block. This iterative refinement process allows the model to progressively focus on pertinent regions of the image.

The motivation for the multi-scale dense attention module is from the clinical scenario. In the clinical scenario of interpreting CBCT scans depicting dental structures, the observer initially perceives the broader features, such as the positioning and dimensions of dental anatomy and associated pathologies; this is depicted by the larger scales in our module. Subsequently, attention is directed towards finer nuances: the contours of individual teeth, subtle variations in bone density, and the integrity of periodontal ligaments, as a portrayal reminiscent of the finer scales. Analogous to this perceptual process, our module progressively refines its focus from coarse to fine details, integrating features from various scales. By synthesizing information across multiple levels of granularity, our model enhances its ability to delineate intricate patterns within CBCT images, thereby contributing to precise diagnosis, treatment planning, and evaluation of dental conditions and interventions.

Unlike the previously mentioned module design that included all the scales, we did not incorporate information from the highest scale into the lower scales. The previous study did not conduct an ablation study to determine the optimal number of scales required to produce the attention map within the module. The bottom block extracts the highest level of features, and including such high-level information in the decoding process could make it difficult to interpret and potentially mislead the learning process. Therefore, in our network, we kept the attention maps obtained from the last four scales and propagated them to all subsequent scales.

### Auto-adapting schema

To address the issue of redundant background information in CBCT images, we incorporated an auto-adapting schema that adjusts the input image volume dynamically during the training process and consequently eliminates irrelevant background information. CBCT images are 3-dimensional and cover the area from the chin to the calvarium, but the jawbone is only a portion of the image, thus making the lesion even smaller compared to other background structures such as teeth and bones. To address the issue of redundant background information in the image, we incorporated an auto-adapting schema that adjusts the input image volume dynamically during the training process therefore eliminating irrelevant background information. The backbone is firstly trained for 100 epochs. Once a preliminary segmentation result is obtained, the input images will be cropped using the predicted labels generated by the same backbone. Specifically, the bounding box of the predicted labels will be obtained by identifying the highlighted area as those pixels are labelled as 1s and others as 0s. The original image will be cropped in the next step using the obtained coordinates. The cropped images are then used as the input images to continue the training process, where the pre-trained backbone used to generate preliminary segmentation results will be trained again with the cropped images. More specifically, given an output label mask $M_{n}$ produced by the network after processing the input image $I_{n}$ in epoch n , the input image $I_{n+1}$ in the next epoch is auto-adjusted using the $M_{n}$ as a reference; if no auto-adapting is applied, the input image in the next epoch would still be the same as the current epoch, as we centralized and crop $I_{n}$ using the label information retrieved from $M_{n}$ , we can obtain $I_{n+1}$ by cropping $I_{n}$ so that the new input image will have less background information and the lesion will be properly centralized to enhance the learning effectiveness of the model further. The label mask generated using input images will be scaled up first and then used to guide the cropping operation for $I_{n+1}$ . We selected the parameters to keep the exact image size between $I_{n}$ and $I_{n+1}$ .

### Size aware loss

We implement the loss function as a combination of multiple losses, the final loss function as:


(1) $$\begin{array}{c} L=\sum_{i=1}^{N}l_{bce}\left(P_{i},Y_{i}\right)+l_{sd}\left(P_{i},Y_{i}\right)\; \end{array}$$

(2) $$\begin{array}{c} l_{sd}\left(P_{i},Y_{i}\right)=\frac{\left|P_{i}-Y_{i}\right|}{\left|Y_{i}\right|}-\frac{2\times \left|P_{i}\cap^{}Y_{i}\right|}{\left|P_{i}\right|+\left|Y_{i}\right|}\; \end{array}$$

Where $P_{i}$ represents the prediction map and $Y_{i}$ represents the ground truth label map. In Equation 1, $l_{bce}$ represents the Binary Cross Entropy Loss function, and $l_{sd}$ represents the Size-aware Dice Loss. As shown in Equation 2, the later fraction represents the original Dice Loss function. The first fraction has a numerator $P_{i}-Y_{i}$ to calculate the size difference between $P_{i}$ and $Y_{i}$ and it is divided by $Y_{i}$ to give the difference ratio. More specifically, we adjust the loss equation to make the dice loss aware of sizes, and we also constrain the range of the loss by setting the size-aware to 1 if we have a huge size ratio to make sure all the losses have a unified scale.

### Implementation

Our OCL-Net was implemented in PyTorch on an NVIDIA GTX 3080 Ti GPU. The total training epoch was set to 200, and the model was trained with Adam optimizer with an initial learning rate of $1\times e^{-4}$ and batch size of 1 . The backbone was implemented following the 3D-UNet architecture (37). All the input images were resized as 128×128×128 . For the auto-adapting schema, the generated label masks are scaled to 256×256×256 , and the image cropping size is set to 128×128×128 . Furthermore, the hyperparameter n was set to 100, which means that the model will first train 100 epochs for the label mask. At the end of the 100^th^ epoch, all the input images will be cropped following the auto-adapting schema, and the adapted input images will be used for the later training processes.

### Evaluation

For quantitative evaluation of the segmentation result, we used the Dice score, Intersection over Union (IoU) and the Area Under the Receiver Operating Characteristic Curve (AUC). The selection of evaluation metrics follows similar settings for segmentation research developed in a clinical context (38, 39).

AUC measures the overall performance of a binary classification model by assessing its ability to distinguish between positive and negative instances based on the ROC curve.

The Dice coefficient is a similarity metric used to measure the overlap between two sets, often used in image segmentation tasks. As shown in Equation 3, $\left|A\cap^{}B\right|$ is the size of the intersection of sets A and B, and |A|,|B| represents the size of sets A and B. IoU measures the overlap between the predicted and ground truth regions in tasks like object detection and segmentation. The equation of IoU is shown in Equation 4, where $\left|A\cup^{}B\right|$ stands for the size of the union of regions A and B .


(3) $$\begin{array}{c} Dice=\frac{2\times \left|A\;\cap^{\;}B\right|}{\left|A\right|+\left|B\right|}\; \end{array}$$

(4) $$\begin{array}{c} IOU=\;\frac{\left|A\;\cap^{\;}B\right|}{\left|A\;\cup^{\;}B\right|}\; \end{array}$$

Since no previous studies were found to be similar to this project, we compared our OCL-Net performance to the backbone UNet and other UNet variants including ResUNet (40) which incorporates residual connections within the architecture and Attention UNet (41) which incorporates attention mechanisms to better focus on the important regions. We chose these models because they have been proven to be robust and can perform well on similar medical imaging tasks. An ablation study to quantitatively compare the contributions from our proposed multi-scale module and the auto-adapting schema is conducted.

To assess the diagnostic assistance ability of our OCL-Net results, we compared the performance of maxillofacial surgeons with and without using the segmentation labels when performing lesion diagnosis on CBCT images and the Welch t-test was conducted to analyze the results obtained from the diagnostic assessment. We selected 3 junior surgeons with less than 3 years of experience in the field, 3 senior surgeons with over 3 years but under 10 years of experience and 1 chief surgeon with over 20 years of experience. From our database of 300 images, we randomly selected 20 images from all 4 diseases, with half of them consisting of original images only and the other half also including the segmentation labels derived from the OCL-Net. Clinicians were asked to go through all the images and perform their diagnosis while recording the time spent and their final decision on the diseases. After this process, each surgeon filled out a questionnaire to provide their opinions of whether the OCL-Net segmentation labels were useful in their diagnostic process.

## Result

### Evaluation of model performance

**Table 1** presents the results of OCL-Net in comparison to UNet, ResUNet and Attention UNet as baselines. An ablation study is also included. The OCL-Net result is the combination of the baseline model with size loss, multi-scale (4) and auto-adapting (S). Our model outperformed the baseline models in every metric including the Dice score by 2%, IoU by 3%, and AUC by 0.5%. Our ablation study shows that the introduction of size loss brought increment across all three metrics. For multi-scaled blocks, four multi-scaled blocks were optimal compared to five blocks, and hence it was selected as the final number of blocks.

**Table 1:** Overview of the OCL-Net performance compared to UNet, ResUNet, Attention UNet and OCL-Net variants (ablations are done based on UNet with the highest result among baseline models) with size loss and using different numbers of multi-scale blocks and different (D) or same (S) image sizes after processing.

	Dice (%)	IoU (%)	AUC (%)
UNet	86.65	78.07	91.81
ResUNet	53.76	41.88	72.11
Attention UNet	80.85	72.92	88.44
+ Size Loss	86.90	79.27	92.01
+ Multi-Scale (5)	86.23	78.05	91.15
+ Multi-Scale (4)	87.45	79.68	**92.61**
+ Auto-Adapting ( D )	87.01	78.63	90.82
+ Auto-Adapting ( S )	88.04	80.48	91.86
OCL-Net	**88.84**	**81.23**	92.37

Note that the bold results represent the best among that metric.

The auto-adapting schema worked well in both image sizes, with 0.3% enhancement in case (D) and 1.3% Dice increase in case (S). The result after upscaling the label masks and trying to keep the same image resolution for all the inputs in case (S) was better with 1% over the experiment result without upscaling in case (D).

**Figure 3:**
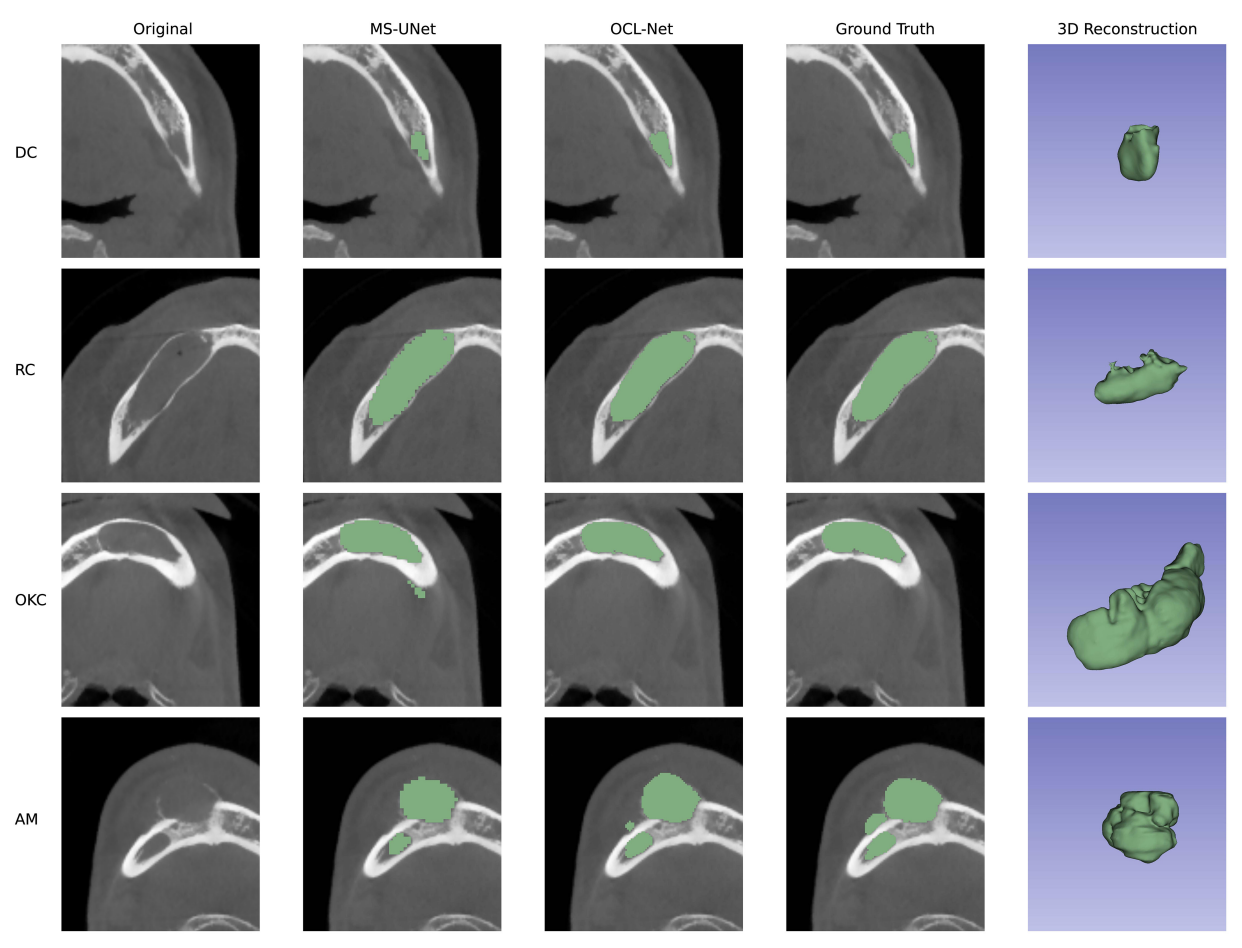
Prediction results compared between UNet with multi-scale and our OCL-Net. The lesion subtypes are shown in rows, ordered from top to bottom as follows: dentigerous cyst, radicular cyst, odontogenic keratocyst and ameloblastoma. Among columns, the first column displays the original images that were cropped using the auto-adapting schema. The second column shows the prediction outcome obtained using U-Net and multi-scale modules, which were manually trimmed to the same size as the others for comparison with the auto-adapting outputs. The third column demonstrates the result segmented by our OCL-Net, combining the multi-scale module and the auto-adapting method. The fourth column depicts the actual ground truth label, and the last column contains the 3D model generated using ground truth.

**Figure 3** displays the original image, prediction results, and 3D reconstruction results generated by 3D Slicer using its build-in function (42). The third column’s prediction results, obtained through OCL-Net, offer a smoother boundary and a more precise outcome compared to the second column, derived from the masks generated by a simple combination of U-Net and multi-scale blocks. In the first two rows, the segmentation masks generated by OCL-Net segmented more pixels around the boundary. In the last two rows, the masks generated by OCL-Net can better locate the lesion without creating false positive segmentation results. We manually crop the images in the second column after segmentation to ensure that all the displayed images having the same size.

### Comparison of clinician performance with assistance from the model

The results of the clinical diagnosis using our OCL-Net are shown in **Table 2A**. The results suggest that within either the original group or the model-assisted group, there were variations in diagnostic accuracy among different participating surgeons. The average accuracy of the chief surgeon (70%) was higher than that of senior surgeons (50%) and junior surgeons (50%). Regardless of whether having assistance with segmentation masks or not, there was no significant impact on the average diagnostic accuracy among all the surgeons. However, in the senior doctors’ group, we observed that the average diagnostic accuracy of Senior 1 and Senior 3 increased from 50% to 65% and 40% to 48%, while Senior 2 dropped from 63% to 53%, respectively. In the junior doctors’ group, the average diagnostic accuracy of participants 2 and 3 had increased from 48% to 58% and 40% to 53%, while participant 1’s accuracy decreased from 63% to 43%, respectively.

**Table 2A:** Diagnostic accuracies obtained from the diagnostic assessment process.

ACC (%)	Chief	Senior 1	Senior 2	Senior 3	Junior 1	Junior 2	Junior 3	AVG	SD
Without Assistance									
DC	0.80	0.50	1.00	0.50	0.70	0.50	0.40	0.63	0.21
RC	0.80	0.80	0.80	0.20	0.80	0.50	0.40	0.61	0.25
OKC	0.60	0.10	0.40	0.40	0.20	0.50	0.20	0.34	0.18
AM	0.60	0.60	0.30	0.50	0.80	0.40	0.60	0.54	0.16
AVG	0.70	0.50	0.63	0.40	0.63	0.48	0.40	0.53	0.12
SD	0.12	0.29	0.33	0.14	0.29	0.05	0.16	0.13	–
With Assistance									
DC	0.80	0.80	0.80	0.30	0.40	0.60	0.50	0.60	0.21
RC	0.67	0.70	0.90	0.80	0.90	0.50	0.40	0.70	0.19
OKC	0.60	0.30	0.20	0.30	0.00	0.30	0.50	0.31	0.20
AM	0.80	0.80	0.20	0.50	0.40	0.90	0.70	0.61	0.25
AVG	0.72	0.65	0.53	0.48	0.43	0.58	0.53	0.56	0.10
SD	0.10	0.24	0.38	0.24	0.37	0.25	0.13	0.17	–

**Table 2B:** Diagnostic time spent shown in 2b obtained from the diagnostic assessment process.

Time (s)	Chief	Senior 1	Senior 2	Senior 3	Junior 1	Junior 2	Junior 3	AVG	SD
Without Assistance									
DC	59.9	142.3	92.0	115.9	109.4	84.7	89.5	99.1	26.3
RC	61.6	132.9	109.2	107.8	106.9	140.8	142.1	114.5	28.1
OKC	49.5	127.7	101.2	113.2	87.1	113.0	105.8	99.6	25.4
AM	54.7	142.8	94.1	92.9	80.2	93.5	87.5	92.2	26.3
AVG	56.4	136.4	99.1	107.5	95.9	108.0	106.2	101.4	23.8
SD	5.5	7.4	7.8	10.3	14.5	24.9	25.3	9.4	–
With Assistance									
DC	50.4	24.8	41.3	39.6	36.5	42.3	45.4	40.0	8.0
RC	59.2	29.7	34.7	53.0	44.2	45.6	55.3	46.0	10.9
OKC	56.1	25.7	49.6	47.0	50.5	48.9	60.7	48.4	11.1
AM	89.8	29.0	60.8	44.6	52.0	50.0	54.9	54.4	18.5
AVG	64.0	27.3	46.6	46.1	45.8	46.7	54.1	47.2	11.0
SD	17.7	2.4	11.3	5.6	7.1	3.5	6.4	6.0	–

A comparison of the diagnostic accuracy for the different diseases between the original group and the model-assisted group is presented in **Table 2A**. The results showed that the chief surgeon was able to maintain a similar diagnostic accuracy for different diseases. However, there were fluctuations in the diagnostic accuracy among senior and junior surgeons. The diagnostic accuracy for RC, DC, and AM was ~60%, while the accuracy for OKC was only ~30%, which is the lowest among all disease types.

**Table 2B** presents the time required for the diagnosis. Without the OCL-Net’s labels, the time needed for our chief surgeon was significantly lower ( P<0.05 ) than that for the senior and junior doctors. However, in the OCL-Net assisted group, the difference in diagnostic times between chief, senior, and junior surgeons was less distinct, except for one senior surgeon who only needed an average time of 27s to diagnose the OCLs. There was no decrease in the time required for the chief surgeon, while senior and junior surgeons experienced a reduction in the average diagnosis time. The average time spent by all three senior surgeons decreased from 114.3s to 39.98s and the average time spent by all three junior surgeons decreased from 103.37s to 49.65s, respectively.

## Discussion

This study demonstrated the effectiveness of a supervised deep-learning method in segmenting odontogenic cystic lesions on CBCT images among four disease subtypes. Our OCL-Net improved in Dice Score (88.84%) and IOU Score (81.23%) compared to other models as shown in **Table 1**. However, the highest AUC score of 92.62% was obtained by combining the U-Net with multi-scale (4). We attribute this improvement to the multi-scale module that enables the model to consider information extracted from different scales thereby improving its ability to distinguish between lesion and background pixels. On the other hand, the auto-adapting method helps the model to concentrate on lesions and eliminate background information, thus leading to accurate delineation of the target region with higher Dice and IoU scores compared to the UNet. Overall, our OCL-Net combines the advantages of both techniques and produces a balanced improvement, achieving the highest Dice and IoU scores and the second-highest AUC score for the OCLs segmentation task. It is unexpected to see the two UNet variants performing worse than the baseline UNet. Since these UNet variants were not designed for CBCT images encompassing both jawbones and the cranial regions, this requirement presented unique challenges to these models. These challenges may arise from the inclusion of redundant background information, compounded by the intricate nature of the bony structures involved. As the amount of lesion pixels is relatively less than the amount of background pixels, simpler architecture like UNet can perform better without overfitting.

Our diagnostic assessment experiment indicates that the highest diagnostic accuracy does not exceed 80%. The chief surgeon shows consistent diagnostic accuracy for various diseases, at approximately 80%. In contrast, the diagnostic rates for senior and junior surgeons are lower, averaging only 50%. The accuracy for diagnosing OKC is the lowest at only 30% where OKC is more likely to be misdiagnosed as DC and AM. The shared imaging characteristics among these three types of lesions are likely responsible for the difficult diagnosis (11). The average diagnostic accuracy increased from 53% to 56% when using our segmentation labels. The improvement in the accuracy may not be due to the segmentation label only, but also because the images in the two experimental groups (with and without the aid of the segmentation labels) are different and therefore have different levels of complexity.

The Welch Two Sample t -test was conducted to compare the average time spent by surgeons diagnosing a disease with and without the aid of segmentation labels. The results reveal a substantial and statistically significant difference between the two groups. Surgeons, on average, took approximately 101.62 seconds to diagnose the disease without segmentation labels, while with the assistance of segmentation labels, the average diagnosis time was significantly shorter at approximately 47.10 seconds. This difference in mean diagnosis times is supported by a high t -statistic of 18.237 and a p-value of $2.2e^{-16}$ , indicating strong evidence that adding segmentation labels leads to a significant reduction in the time required for diagnosis. The 95 per cent confidence interval suggests that the true difference in the diagnosis times is likely to be higher than 49.59 seconds. Despite the segmentation labels contributing to diagnostic efficiency, during the normal diagnostic process, surgeons are unlikely to manually segment the lesion, as labelling an accurate segmentation label may take a longer time than the diagnosis. Automatically generating segmentation masks would help a lot in diagnostic efficiency in real clinical practice.

In our ablation study, regarding the parameter selection for the multi-scale module, we chose to remove the multi-scaled block that is linked to the bottom block. The primary objective of this block is to extract the features from the highest scale. Since our lesions are relatively small compared to the background, the highest-level information could be influenced heavily by unrelated information possessed in the background. Conveying such high-level information to the model and including it in the decoding process may misguide the learning process of the model. Hence, we decided to decrease the number of multi-scale blocks, and our experimental findings have validated our decision with dice score improvement and a smoother training process. As the original multi-scaled technique implemented a paired supervision method by comparing the SoftMax output of every multi-scale block to the down-sampled ground truth map. In the initial experiment setup, we followed their method of combining all five losses, but the loss for the fifth scale, which is the bottom block, never converged. This outcome is consistent with our claim, proving that the highest level of information may impede the training process of the model.

The upscaling process within our auto-adapting schema was designed to retain the same image resolution as the original input image. The choice of input image resolution can significantly impact the performance of deep learning models. The resolution of the input images determines the amount of detail that the model can capture, and the computational complexity of the model. On one hand, using high-resolution images can improve the model’s ability to capture fine-grained details, which can lead to better performance. On the other hand, using low-resolution images can reduce the computational complexity of the model and speed up the training process, but at the cost of potentially sacrificing some information. A study by Thambawita et al. investigated the effect of different image resolutions on the performance of CNNs on a public dataset and found that using higher-resolution images led to better performance (43). In our experiment, we upsized the trained segmentation output and used it as a reference to centralize the input image. The upscaling process does not need to be precise because only the center of the lesion should be acquired. Finally, the nearest up-sampling algorithm was implemented and as seen in **Table 1**, the auto-adapting method resulted in a higher performance with the upscaling process and produced a higher result (Dice 88.04%, IoU 80.48%, AUC 91.86%) compared to cropping the input image (Dice 87.01%, IoU 78.63%, AUC 90.82%).

Introducing the size-aware loss function represents an advancement in our approach to semantic segmentation. Unlike conventional Dice Loss, which solely focuses on the pixel-wise agreement between prediction and ground truth, our size-aware formulation accounts for differences in lesion sizes by evaluating the ratio of differences and intersections between prediction and ground truth masks relative to the ground truth size. This adjustment makes the loss function aware of the size disparities among the lesions and ensures that the loss is within a specific range, thus promoting stable and consistent model training. Integrating the size-aware loss into our training pipeline yielded promising results, as evidenced by the increase across different metrics (+0.25% in Dice, +1.2% in IoU and +0.2% in AUC). By explicitly considering lesion sizes during training, our model demonstrated improved sensitivity to variations in lesion morphology, leading to enhanced segmentation accuracy across different image samples. This highlights the effectiveness of our approach in mitigating the challenges posed by size discrepancies, which are common in medical imaging datasets.

We also applied the t -test to our model compared to the baseline. The results of the t -tests yielded insights into the comparative performance of OCL-Net compared to UNet. The t-statistic for Dice is approximately -2.384, with a corresponding p-value of 0.024. This suggests a statistically significant difference in the Dice scores between the two models. Specifically, the negative t-statistic implies that the mean Dice score of the baseline model is lower than that of the OCL-Net. These findings suggest that the OCL-Net offers superior segmentation accuracy and overlap with the ground truth labels, as indicated by higher Dice scores compared to the baseline UNet.

The generated segmentation masks can serve as invaluable tools with multifaceted clinical relevance. They enable precise diagnosis by accurately delineating the extent of the lesions which are crucial for treatment planning. With lesion segmentation masks, dentists can visualize the lesion size and its location, and this can aid in selecting appropriate clinical interventions. Additionally, these masks allow for ongoing monitoring of lesion progression, facilitating assessment of treatment efficacy and adjustments as needed. Apart from potential clinical applications, visual aids from the segmentation masks can be used to enhance patient education and promote a better understanding of oral health conditions and treatment options.

This study focused on a single lesion and a single pathological type of lesion, meaning that within each image, only one type of lesion was considered. We demonstrated that our method was capable of learning the characteristics of these lesions and being able to segment them. It’s important to note that there is a possibility for a small number of patients to have multiple types of lesions simultaneously, and as future work, we will explore the extension of our method to cater for multiple lesions. To improve or expand the segmentation task for OCLs using CBCT images in the future, we will investigate incorporating additional subtypes of the jaw lesions which were not selected in this study because there weren’t enough patients. Including more subtypes will result in a more diverse and extensive dataset. Which can help with the robustness and generalizability of the segmentation system.

In conclusion, this study demonstrated that our OCL-Net achieved > 88% Dice Score in segmentation results for OCL using CBCT images. In our experiments, OCL-Net was able to improve the speed and accuracy of diagnosis therefore reducing the workload of clinical doctors. We suggest that the possibility of applying supervised deep learning segmentation methods in clinical settings is dependent on extra downstream research, such as utilizing the segmentation system to automatically crop the lesions from the original images and use it to help with other lesion analysis and volume characteristics research in the future.

## Data availability statement

The datasets presented in this article are not readily available because we want to ensure that the interests of patients are upheld. Requests to access the datasets should be directed to Zimo Huang, zimo.huang@sydney.edu.au.

## Ethics statement

The studies involving humans were approved by Institutional Medical Ethics Committee of the Stomatological Hospital of Wuhan University (2020-B22). The studies were conducted in accordance with the local legislation and institutional requirements. Written informed consent for participation was not required from the participants or the participants’ legal guardians/next of kin in accordance with the national legislation and institutional requirements. Written informed consent was obtained from the individual(s) for the publication of any potentially identifiable images or data included in this article.

## Author contributions

ZH: Conceptualization, Data curation, Formal analysis, Investigation, Methodology, Validation, Visualization, Writing – original draft, Writing – review & editing. BL: Data curation, Resources, Validation, Writing – review & editing. YC: Data curation, Project administration, Resources, Validation, Writing – review & editing. JK: Conceptualization, Formal analysis, Methodology, Supervision, Validation, Writing – review & editing.
